# AEBP1 Is One of the Epithelial-Mesenchymal Transition Regulatory Genes in Colon Adenocarcinoma

**DOI:** 10.1155/2021/3108933

**Published:** 2021-12-12

**Authors:** Dandan Li, Zhi Liu, Xiaorong Ding, Zhensheng Qin

**Affiliations:** ^1^Department of Anorectal Surgery, The Affiliated Huaian No.1 People's Hospital of Nanjing Medical University, China; ^2^Department of Oncology, The Affiliated Huaian No.1 People's Hospital of Nanjing Medical University, China

## Abstract

Epithelial-mesenchymal transition (EMT) is involved in various tumor processes, including tumorigenesis, tumor cell migration and metastasis, tumor stemness, and therapeutic resistance. Therefore, it is important to identify the genes most associated with EMT and develop them as therapeutic targets. In this work, we first analyzed EMT hallmark gene expression profiles among 10,535 pan-cancer samples from The Cancer Genome Atlas (TCGA) and divided them into EMT high and EMT low groups according to the metagene scores. Then, we identified 12 genes that were most associated with high EMT metagene score (*R* > 0.9) in 329 colon adenocarcinoma (COAD) patients. Among them, only 4 genes (AEBP1, KCNE4, GFPT2, and FAM26E) had statistically significant differences in prognosis (*P* < 0.05). Next, we selected AEBP1 as a candidate and showed that AEBP1 mRNA levels and EMT biomarkers strongly coexpressed in 329 COAD samples. In addition, AEBP1 was highly expressed and associated with poor clinical outcomes and prognosis in COAD patients. Finally, to explore whether AEBP1-mediated EMT was related to the tumor microenvironment (TME), we examined AEBP1 expression levels at the single-cell levels. Our results showed that AEBP1 levels were extremely high in tumor-associated fibroblasts, which may induce EMT. AEBP1 expression was also positively correlated with the expression of fibroblast biomarkers and also with EMT metascores, suggesting that AEBP1-mediated EMT may be associated with the stimulation of fibroblast activation. Therefore, AEBP1 may be a promising target for EMT inhibition, which reduces cancer metastasis and drug resistance in COAD patients.

## 1. Introduction

Colon adenocarcinoma (COAD) is characterized by late onset, rapid tumor progression, and heterotopic metastasis, and it is the third leading cause of cancer-related deaths in the world [1]. Radiotherapy and chemotherapy are the choices of treatment for resectable and advanced COAD [2, 3]. Despite the application of targeted drugs and immunosuppressive agents that extend the survival of patients, there is still a need to overcome metastasis and treatment resistance in COAD.

EMT is a cellular process by which cells lose their epithelial characteristics and gain mesenchymal properties [4]. EMT has been involved in a variety of tumor characteristics, including tumorigenesis, malignant progression, tumor cell migration and metastasis, blood intravasation, tumor stemness, and therapeutic resistance [4–6]. The evidence is overwhelming, on the other hand, that cancer progression and metastasis initiate in cells with the majority of stem cell characteristics, termed cancer stem cells (CSCs) [7]. CSCs are known to be able to self-renew and divide asymmetrically. These cells contribute to tumorigenesis, expansion, resistance, metastasis, and recurrence after treatment [8]. The differences between CSCs and non-CSCs may mainly attribute to the cell biological events involved in EMT [9, 10]. Increased evidence suggests a strong association between EMT and the production of CSCs [11].

EMT involves a variety of signaling pathways that alter the expression of genes by regulating major transcription factors such as Snail, Twist, and ZEB, which increase the expression of mesenchymal cell markers and decrease epithelial cell markers, ultimately transforming the epithelial cell phenotype into mesenchymal stem cells [12, 13]. Snails represent a ZINC finger transcription factor family with participation in different molecular mechanisms. This family includes three members, known as Snail1 (Snail), Snail2 (Slug), and Snail3 (Smuc) [14, 15]. Among them, the most important member is Snail1, which holds essential roles in the induction of migration and metastasis of cancer cells by EMT. Twist, the second effective transcription factor in EMT, consists of two members, namely, Twist-1 (Twist) and Twist-2 (Dermo-1) [16]. In previous studies regarding Twist-1, it was reported to play key functions in cancer metastasis, angiogenesis, and stem cells. However, little information is available on the molecular basis of Twist-2 [17]. Lastly, the zinc finger E-box binding homology box (ZEB) is known as the third major transcription factor of EMT and consists of two members, ZEB1 and ZEB2 [18]. It has been shown that ZEB1 and ZEB2 contribute to the metastatic properties of cancer cells by binding to the E-box sequence of the E-cadherin promoter and suppressing the expression of E-cadherin [18].

Adipocyte enhancer binding protein 1 (AEBP1) was initially considered a transcriptional suppressor of the adipose P2 (aP2) gene in preadipocytes [19]. With a high expression in macrophages, AEBP1 has been demonstrated to stimulate the production of multiple inflammatory mediators, including interleukin 6 (IL-6), monocyte chemotactic protein 1 (MCP-1), and tumor necrosis factor ɑ (TNF-ɑ) [20]. Recent studies have demonstrated that AEBP1 may play vital roles in promoting carcinogenesis. AEBP1 has been implicated in the development and progression of a variety of tumors, including gastric cancer [21], colon cancer [22], and glioblastoma [23].

It is very crucial to find the genes most associated with EMT and to incorporate them into cancer therapeutic strategies for the reduction of cancer metastasis and drug resistance. In this study, we identified one gene most relevant to EMT hyperactivation and explored the roles of its expression on clinical outcomes and prognosis in COAD patients.

## 2. Materials and Methods

### 2.1. Bioinformatics Analysis

Raw gene count expression profiles for pan-cancer and COAD samples were downloaded from The Cancer Genome Atlas (TCGA) database (http://tcga.xenahubs.net). After filtering low-expressing genes and samples of poor RNA quality, gene counts of 10,535 patients were normalized using TPM (transcripts per million reads) methods and corrected for overdispersion. EMT scores were calculated across all patients though 200 genes using the gene set downloaded from MSigDB (https://www.gsea-msigdb.org/gsea/msigdb). We calculated a metagene 0 score as low EMT expression and >1 score as high EMT expression. We also calculated in total 329 COAD samples from the TCGA database to find the most correlating gene (*R* > 0.9) based on the EMT metagene score.

### 2.2. Correlation Analysis

AEBP1 expression, the levels of EMT-related genes (SNAI1, SNAI2, TWIST1, ZEB1, ZEB2, CDH1, CDH2, VIM, MMP2, MMP3, MMP9, KRT8, and TJP1), and the expression of fibroblasts biomarkers (ACTA2, PALLD, PDPN, P4HA3, MMP11, and FAP) were obtained from 329 COAD samples in the TCGA dataset. Pearson correlation coefficient tests were used to estimate the relationship between AEBP1 expression and other genes.

### 2.3. AEBP1 mRNA Expression in COAD Samples

We used UALCAN (http://ualcan.path.uab.edu) resource to explore AEBP1 expression levels in normal and tumors in COAD patients generated from TCGA database [24]. We also investigated the expression of AEBP1 based on individual cancer stages and nodal metastasis status. Pathologic nodal metastasis was descripted as follows: N0, no regional lymph node metastasis; N1, metastases in 1 to 3 axillary lymph nodes; N2, metastases in 4 to 9 axillary lymph nodes; and N3, metastases in 10 or more axillary lymph nodes.

### 2.4. AEBP1 Protein Expression in COAD Samples

We used Clinical Proteomic Tumor Analysis Consortium (CPTAC) Confirmatory/Discovery dataset to check AEBP1 protein levels [25]. In the CPTAC database, the protein expression for colon cancer, breast cancer, ovarian cancer, clear cell renal cell carcinoma, and uterine corpus endometrial carcinoma is available. In this study, we explored AEBP1 protein expression in tumor and normal tissues in COAD samples and the relationship between AEBP1 levels and TNM stages.

### 2.5. Single-Cell Analysis

Tumor Immune Single-Cell Hub (TISCH) (http://tisch.comp-genomics.org/) is used to investigate AEBP1 expression in tumor microenvironment at single-cell resolution. TISCH is a scRNA-seq database focusing on tumor microenvironment (TME). TISCH provides detailed cell type annotations at the single-cell levels, enabling the exploration of TME across different cancer types [26]. In this dataset, there are mainly three cell types, including immune cells, stromal cells, and malignant cells. Immune cells include B cells, conventional CD4 T cells, CD8 T cells, exhausted CD8 T cells, dendritic cells, plasmacytoid dendritic cells, plasma cells, mast cells, monocytes or macrophages, natural killer cells, neutrophils, proliferating T cells, and regulatory T cells. Stromal cells consist of endothelial, fibroblasts, myofibroblasts, and other cells.

## 3. Results

### 3.1. The most Relevant Gene Candidates for EMT in COAD Were Identified

**Figure 1:**
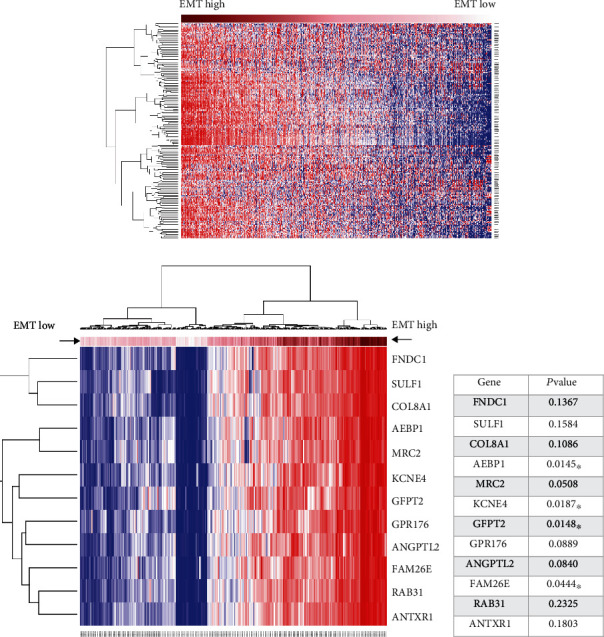
The most relevant gene candidates for EMT in COAD were identified. (a) EMT hallmark gene expression profiles among 10,535 clinical pan-cancer samples from TCGA database were calculated by meta-gene scores (0 - >1, low to high EMT) though 200 genes using hallmark gene sets (https://www.gsea-msigdb.org/gsea/msigdb). The heatmap showed the 200 EMT gene expression pattern based on EMT metagene scores, and the groups were divided into EMT high and EMT low groups according to the metagene scores. (b) The heatmap showed 12 genes that were most associated with high EMT metagene score (*R* > 0.9) in 329 COAD patients. (c) The *P* values of the 12 genes were calculated, and only 4 genes (AEBP1, KCNE4, GFPT2, and FAM26E) had statistical differences (*P* < 0.05). ^∗^*P* < 0.05.

To identify the genes most associated with EMT, we first analyzed EMT hallmark gene expression profiles among 10,535 clinical pan-cancer samples from TCGA database. We calculated metagene scores (0 - >1, low to high EMT) through 200 genes using hallmark gene sets (https://www.gsea-msigdb.org/gsea/msigdb). Heatmap showed the expression pattern from the 200 EMT genes based on EMT metagene scores; then, the groups were divided into EMT high and EMT low groups according to the metagene scores (Figure 1(a)). These 200 genes were all highly expressed in the EMT high score group, indicating that the EMT high score could provide a good representation of the EMT pathway being activated (Figure 1(a)). Next, we discovered the genes associated with EMT based on EMT metagene scores and listed 12 most correlating genes with EMT (*R* > 0.9) in 329 patients with COAD (Figure 1(b)). We further used Cox proportional-hazards radio to filter the prognostic-related genes. Among these 12 genes, only 4 genes (AEBP1, KCNE4, GFPT2, and FAM26E) had statistical differences (*P* < 0.05) (Figure 1(c)).

### 3.2. AEBP1 Is Highly Associated with EMT in COAD Patients

**Figure 2:**
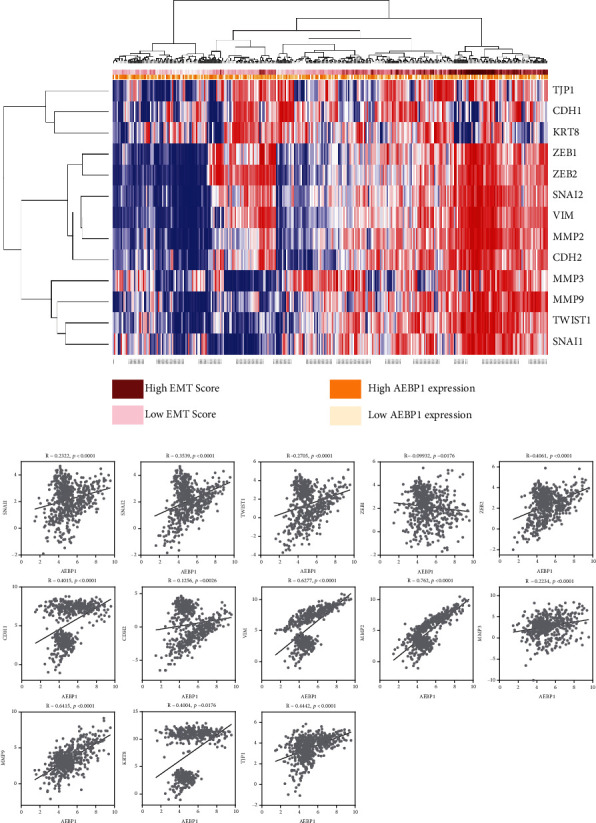
AEBP1 is highly associated with EMT in COAD patients. (a) Heatmap analysis showed AEBP1 expression and the levels of EMT biomarkers (SNAI1, SNAI2, TWIST1, ZEB1, ZEB2, CDH1, CDH2, VIM, MMP2, MMP3, MMP9, KRT8, and TJP1) in 329 COAD patients. The high mRNA levels of AEBP1 were positively associated with EMT metagene scores and the expression of EMT-related genes. (b) Correlation between AEBP1 expression and the levels of EMT biomarkers (SNAI1, SNAI2, TWIST1, ZEB1, ZEB2, CDH1, CDH2, VIM, MMP2, MMP3, MMP9, KRT8, and TJP1) in 329 COAD patients.

We next selected AEBP1 as the candidate of its regulation of EMT and its roles in COAD. Since primary analyses suggested that the EMT gene set was significantly enriched in the high levels of the AEBP1 group, we therefore explore whether AEBP1 expression levels have linked to EMT biomarkers. To better understand the correlation between the AEBP1 expression and the levels of EMT-activated genes, we investigated both AEBP1- and EMT-related genes (SNAI1, SNAI2, TWIST1, ZEB1, ZEB2, CDH1, CDH2, VIM, MMP2, MMP3, MMP9, KRT8, and TJP1) levels in EMT activation status (Figure 2(a)). Our data showed that high mRNA levels of AEBP1 were positively associated with EMT metagene scores, which presented the similar trend as the EMT-related gene expression, indicating that there might be a positive correlation between AEBP1 mRNA levels and the expression of EMT-activated genes (Figure 2(a)). To confirm our hypothesis, we explored the coexpression of AEBP1 and EMT biomarkers. The results showed that the mRNA levels of AEBP1 and EMT biomarkers greatly correlated in 329 COAD samples in the TCGA database (Figure 2(b)). These results confirmed that AEBP1 is strongly correlated with EMT in COAD patients.

### 3.3. AEBP1 Is Highly Expressed and Associated with Poor Clinical Outcomes in COAD Patients

**Figure 3:**
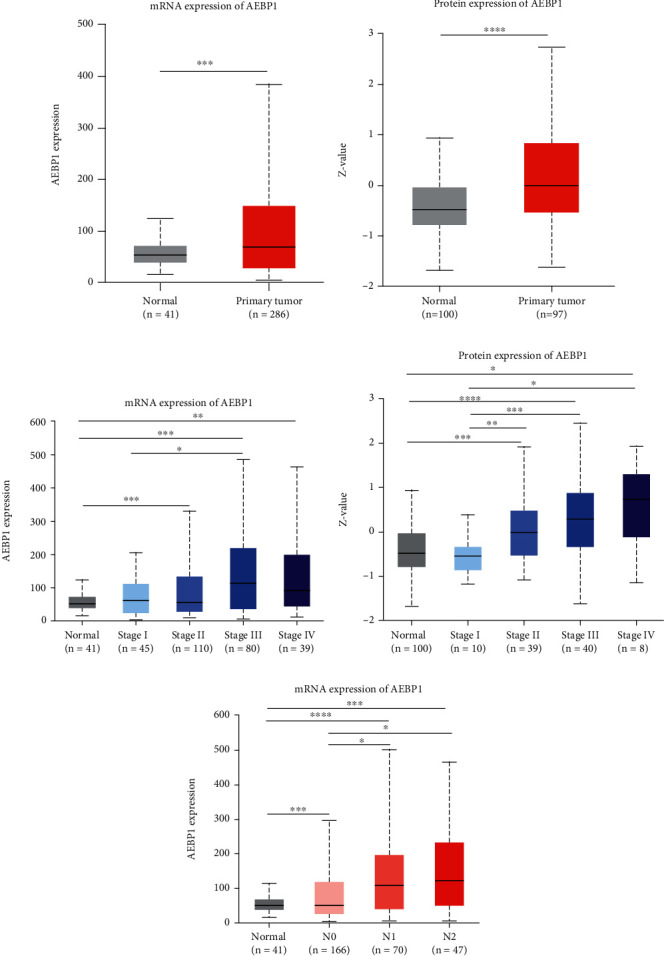
AEBP1 is highly expressed and associated with poor clinical outcomes in COAD patients. (a) The mRNA expression of AEBP1 in 41 normal tissues and 286 COAD tumors from TCGA dataset generated by UALCAN. (b) The protein levels of AEBP1 in 100 normal tissues and 97 tumors from CPTAC database generated by UALCAN. (c) The mRNA expression of AEBP1 in different TNM stages from TCGA dataset. (d) AEBP1 protein levels in different TNM stages from CPTAC database. (e) The mRNA expression of AEBP1 in different lymph node metastasis status from TCGA database. ^∗^*P* < 0.05, ^∗∗^*P* < 0.01, ^∗∗∗^*P* < 0.001, and ^∗∗∗∗^*P* < 0.0001.

To explore the effects of AEBP1 on clinical patients, we first investigated the expression levels of AEBP1 among normal and tumor samples in cancer patients in the TCGA database. The result showed that the mRNA expression of AEBP1 was significantly upregulated in almost all clinical cancer tissues compared to normal tissues, including COAD (Supplement Figure 1 and Figure 3(a)). Similarly, we also investigated the AEBP1 protein levels in the CPTAC dataset and showed the overexpression of AEBP1 in COAD samples (Figure 3(b)).

To better understand the relationship between AEBP1 expression and clinical features, we assessed AEBP1 levels in different TNM stages and nodal metastasis status, in which higher TNM stages and more nodal metastasis exhibit faster tumor progression and greater metastatic ability. We observed that there were higher mRNA expression levels of AEBP1 in all stages (stage I, stage II, stage III, and stage IV) of tumor samples than in normal samples (Figure 3(c)). Moreover, a higher TNM stage (stage III) showed stronger AEBP1 expression than a lower stage (stage I) of tumor samples (Figure 3(c)), suggesting that AEBP1 mRNA expression levels were correlated with TNM stages of COAD patients. Similarly, AEBP1 protein levels were significantly related to TNM stage in COAD patients (Figure 3(d)). In addition, the increased AEBP1 expression was obviously correlated with more metastatic lymph nodes (Figure 3(e)). Taken together, AEBP1 is significantly upregulated and related to poor clinical features in COAD patients.

### 3.4. AEBP1 Is Correlated with Poor Clinical Prognosis in COAD Patients

**Figure 4:**
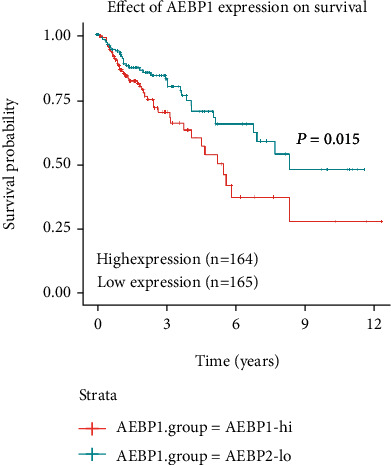
AEBP1 is correlated with poor clinical prognosis in COAD patients. The correlation between AEBP1 expression and OS in COAD patients in TCGA database. Kaplan-Meier analysis suggested that the high expression of AEBP1 was related to poor OS in 329 COAD patients (*P* < 0.05).

We next investigated the relationship between AEBP1 levels and the survival of patients with COAD. Kaplan-Meier analysis suggested that the high expression of AEBP1 was related to short overall survival (OS) in 329 COAD patients (*P* < 0.05) (165 samples with low AEBP1 expression and 164 samples with high AEBP1 expression) in the TCGA database (Figure 4). These results suggested that higher expression of AEBP1 was associated with worse prognosis in patients with COAD.

### 3.5. AEBP1 Is Mainly Expressed in Fibroblasts

We have demonstrated that AEBP1 was greatly associated with the EMT signaling pathway, AEBP1 was highly expressed, and that high expression of AEBP1 was correlated with poor patient pathological characteristics and prognosis in COAD patients. However, we still do not know how AEBP1 regulates EMT.

**Figure 5:**
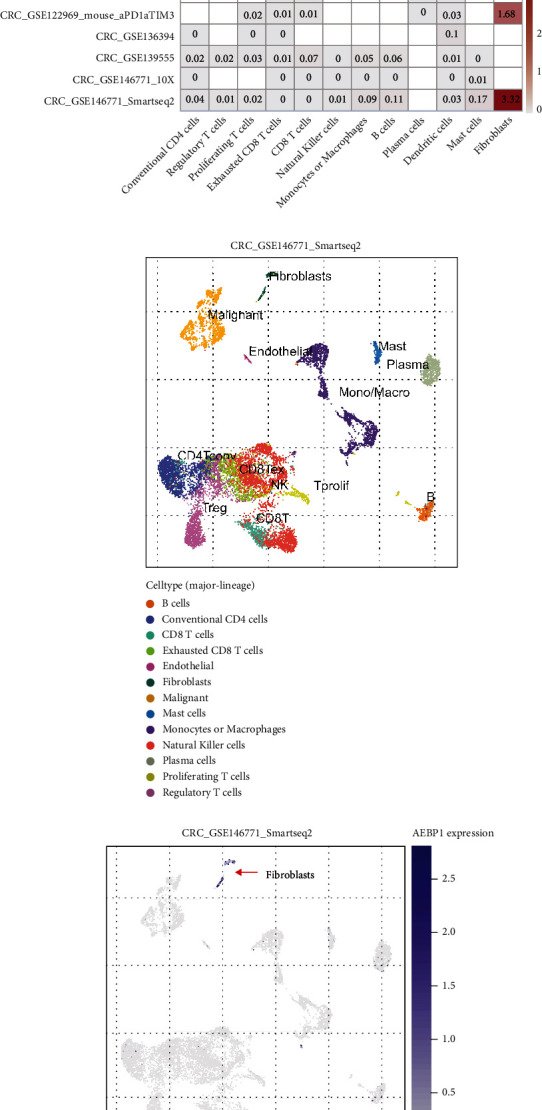
AEBP1 is mainly expressed in fibroblasts. (a) Heatmap analysis showed average expression of AEBP1 in different cell types across the TISCH dataset in all cancers. (b, c) Heatmap analysis showed average expression of AEBP1 in different cell-types across TISCH dataset in COAD. (d–f) The distribution of AEBP1 in different cell types was analyzed in the GSE146771_Smartseq2 dataset. (g, h) The distribution of AEBP1 and EMT biomarkers (SNAI1, SNAI2, TWIST1, ZEB1, and ZEB2) in different cell types was analyzed in the GSE146771_Smartseq2 dataset.

The TME consists of extracellular matrix (ECM), tumor-associated fibroblasts, myofibroblasts, immune cells, and soluble factors associated with tumor development and metastasis [27]. In the TME, EMT can be induced among tumor cells by autocrine or paracrine secretion of some mediators such as growth factors, cytokines, and extracellular matrix proteins [28, 29]. In cancer cells, tumor-associated fibroblasts are able to induce EMT in tumor cells [30]. Therefore, to further explore whether AEBP1-mediated EMT was associated with the TME, we examined in the TISCH database, a single-cell hub, in which cell populations AEBP1 was mainly expressed. Firstly, the levels of AEBP1 were analyzed of single-cell subpopulations across all tumors, including immune cells, tumor cells, and stromal cells. Interestingly, AEBP1 was expressed in malignant cells and stromal cells, especially predominantly in stromal cells, which was consistent in almost all tumors, as well as in COAD samples (Figures 5(a) and 5(b)). The tumor-associated stromal cells mainly include endothelial, fibroblasts, and myofibroblasts. We next examined which stromal cell component was the AEBP1 high-expressing cells in COAD. The results showed that fibroblasts were the major expressing cells (Figure 5(c)). We further explored one of the COAD GSE dataset (GSC_GSE146771_Smartseq2) (Figure 5(d)); AEBP1 levels were extremely highly expressed in fibroblasts (Figures 5(e) and 5(f)).

Next, we explored the expression of AEBP1 and EMT biomarkers (SNAI1, SNAI2, TWIST1, ZEB1, and ZEB2) in different immune cells. AEBP1 and SNAI1, SNAI2, TWIST1, ZEB1, and ZEB2 showed similar expression levels in cell types (Supplement Figure 2). Both of AEBP1- and EMT-related genes were predominantly expressed in stromal cells and, in particular, mainly in fibroblasts (Figures 5(g) and 5(h)).

### 3.6. AEBP1 Expression Is Highly Related to Biomarkers of Tumor-Associated Fibroblasts

We have found that AEBP1 expression was highly expressed in fibroblasts. We further investigated whether AEBP1 expression was linked to tumor-associated fibroblasts biomarkers.

**Figure 6:**
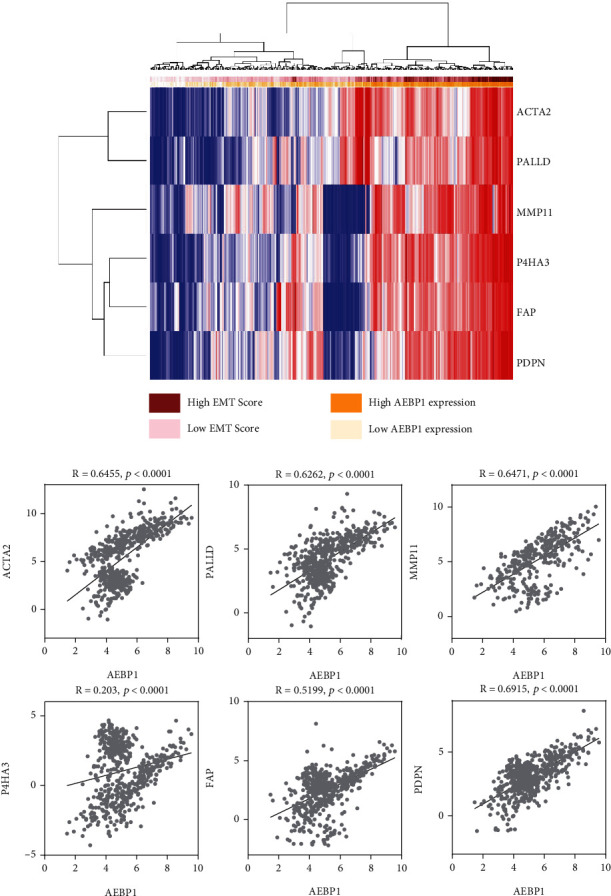
AEBP1 expression is highly related to biomarkers of tumor-associated fibroblasts. (a) Heatmap analysis showed AEBP1 expression and the levels of tumor-associated fibroblasts biomarkers (ACTA2, PALLD, MMP11, P4HA3, FAP, and PDPN) and EMT metascores in 329 COAD patients. (b) Coexpression of AEBP1 expression and fibroblast biomarkers (ACTA2, PALLD, MMP11, P4HA3, FAP, and PDPN) in 329 COAD patients.

Several molecular markers have been used to identify continuously activated fibroblasts, for example, *α*-smooth muscle actin (*α*-SMA) [31] and cytoskeletal protein (palladin) [32]. Recently, several new molecular markers have been reported, including mucin-type protein (podoplanin), matrix metalloproteinase (stromelysin-3), collagen prolyl 4-hydroxylase (P4H), and fibroblast activation protein (FAP), which are associated with the activation of fibroblasts [33]. We then verified our suspicions to check the coexpression of AEBP1 and fibroblast biomarkers, including ACTA2 (*α*-SMA), PALLD (palladin), PDPN (podoplanin), P4HA3, MMP11 (stromelysin-3), and FAP in 329 COAD samples in the TCGA dataset. Interestingly, AEBP1 expression was positively correlated with the expression of fibroblast biomarkers and also with EMT metascores (Figure 6(a)). AEBP1 was extremely coexpressed with fibroblast biomarkers ACTA2 (*R* = 0.6455, *P* < 0.0001), PALLD (*R* = 0.6262, *P* < 0.0001), MMP11 (*R* = 0.6471, *P* < 0.0001), P4HA3 (*R* = 0.203, *P* < 0.0001), FAP (*R* = 0.5199, *P* < 0.0001), and PDPN (*R* = 0.6915, *P* < 0.0001) (Figure 6(b)). These results suggested that AEBP1-mediated EMT may be associated with promoting fibroblast activation.

## 4. Discussion

A high incidence and mortality has been shown in patients with colon cancer [1]. Tumor invasion and metastasis are the underlying causes of treatment failure and lack of efficacy in colon cancer patients [34]. In the development of tumor malignancy, EMT is known to lead to a loss of interadhesion between tumor cells, resulting in an enhanced ability to invasion and migration [35]. Therefore, to improve the clinical prognosis of patients with colon cancer, it is critical to find effective and safe blockers of EMT activation to inhibit tumor progression in colon cancer.

We identified the most relevant genes for EMT activation of 329 clinical samples with COAD from the TCGA database. They are AEBP1, KCNE4, GFPT2, FPT2, and FAM26E. AEBP1 is an important transcriptional repressor involved in the regulation of key biological processes, including inflammation, mammogenesis, and adipogenesis [36]. Increasing studies have shown that AEBP1 is a potential oncogene for different types of cancers. It is aberrantly expressed in various malignancies, including glioblastoma, bladder cancer, breast cancer, colon cancer, ovarian cancer, gastric cancer, and skin cancer [36–38]. Here, we demonstrated that similar results were found with the overexpression of AEBP1 in clinical COAD samples and highly correlated with poor pathological features. In addition to aberrant expression in tumor tissues, AEBP1 also exhibited poor prognostic survival in COAD. Therefore, targeting AEBP1, one of the genes most associated with EMT activation in COAD, may be an effective agent for EMT inhibition.

Currently, several reports suggest that AEBP1 regulates tumor progression through the following mechanisms. AEBP1 plays a role in tumorigenesis via activation of the NF-*κ*B pathway in cancer cells [21, 22]. AEBP1 restrains childhood acute lymphoblastic leukemia (ALL) through a p53-dependent pathway [39]. AEBP1 is able to act on glioma stem-like cells (GSCs) mediated by PI3K/AKT signaling, which is involved in the initiation and maintenance of tumor cells [40]. In this study, we have greatly demonstrated the correlation between AEBP1 and EMT activation. However, whether and how AEBP1 regulates EMT is still unknown. To address this shortcoming, we will confirm the regulatory association between AEBP1 and EMT *in vivo* and *in vitro* experiments in the future to better understand their cancer-promoting mechanisms.

Two factors can induce EMT in tumor cells, including growth factors and the TME. Early studies found that hepatocyte growth factor (HGF) promotes EMT in tumor cells by altering the expression of E-cadherin on the surface of tumor cells [41]. It was found in later studies that transforming growth factor *β* (TGF-*β*), insulin-like growth factor (IGF), fibroblast growth factor (FGF), and epithelial growth factor (EGF) can also bind to the corresponding membrane receptors and activate their downstream signaling pathways, thus inducing EMT in tumor cells [42]. In the TME, EMT can be induced among tumor cells by the secretion of some mediators such as growth factors, cytokines, and extracellular matrix proteins [28, 29]. In cancer cells, tumor-associated fibroblasts could trigger EMT in cancer cells, and the TME could initiate and maintain EMT [43]. Here, we showed that AEBP1 levels were highly expressed in tumor-associated fibroblasts, which may induce EMT. Moreover, AEBP1 expression was positively correlated with the expression of fibroblast biomarkers and also with EMT metascores, suggesting that AEBP1-mediated EMT may be associated with the facilitation of fibroblast activation.

There remain several questions to the current work. First, several other genes most associated with EMT (KCNE4, GFPT2, and FAM26E) (Figures 1(b) and 1(c)) may also regulate EMT activation. We will further explore whether they are also aberrantly expressed in COAD and if their expression levels also have effects on the survival of COAD patients. In addition, our finding suggested that AEBP1 was mainly expressed in tumor-associated fibroblasts and positively associated with fibroblast activation. However, it is unknown whether AEBP1 expression in fibroblasts is exclusively correlated with EMT activation, and what the exact mechanism of how AEBP1 mediates EMT could be. We will follow up with more experiments to further validate our suspicions in the future.

## Data Availability

The data used to support the findings of this study are available from the corresponding author upon request.

## Supplementary Materials

Supplementary 1 Supplement Figure 1: AEBP1 is highly expressed in patients with many cancer types. AEBP1 expression in tumors and normal tissues in many cancer types from TCGA dataset generated by UALCAN.

Supplementary 2 Supplement Figure 2: both of AEBP1 and EMT-related genes were predominantly expressed in fibroblasts. The distribution of AEBP1 and EMT biomarkers (SNAI1, SNAI2, TWIST1, ZEB1, and ZEB2) in different cell types was analyzed in the GSE146771_Smartseq2 dataset.
