# Supplementary figures and images for: AEBP1 Is One of the Epithelial-Mesenchymal Transition Regulatory Genes in Colon Adenocarcinoma
Source: Biomed Res Int. 2021 Dec 12;2021:3108933. doi: 10.1155/2021/3108933 (PMC8685759; doi:10.1155/2021/3108933)

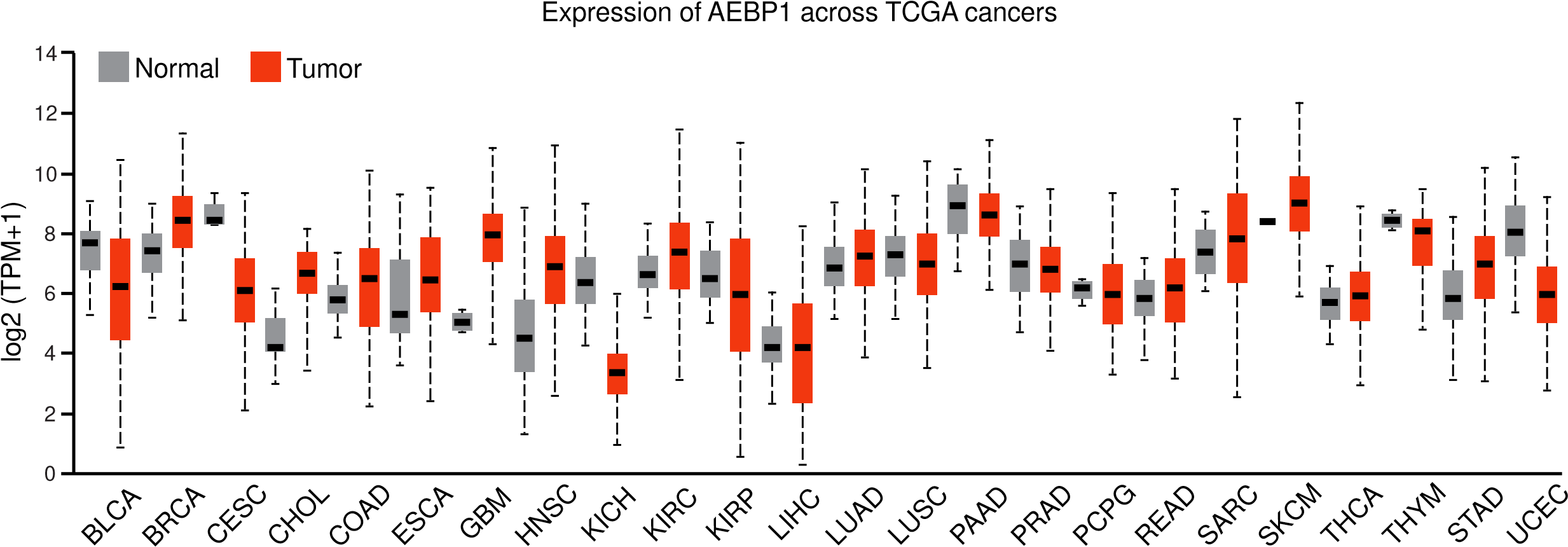


Supplement Figure 1

Supplement: Supplementary 1 — Supplement Figure 1: AEBP1 is highly expressed in patients with many cancer types. AEBP1 expression in tumors and normal tissues in many cancer types from TCGA dataset generated by UALCAN. [file 3108933.f1.doc]
