# Supplementary material for: AEBP1 Is One of the Epithelial-Mesenchymal Transition Regulatory Genes in Colon Adenocarcinoma
Source: Biomed Res Int. 2021 Dec 12;2021:3108933. doi: 10.1155/2021/3108933 (PMC8685759; doi:10.1155/2021/3108933)
Supplement: Supplementary 2 — Supplement Figure 2: both of AEBP1 and EMT-related genes were predominantly expressed in fibroblasts. The distribution of AEBP1 and EMT biomarkers (SNAI1, SNAI2, TWIST1, ZEB1, and ZEB2) in different cell types was analyzed in the GSE146771_Smartseq2 dataset. [file 3108933.f2.doc]

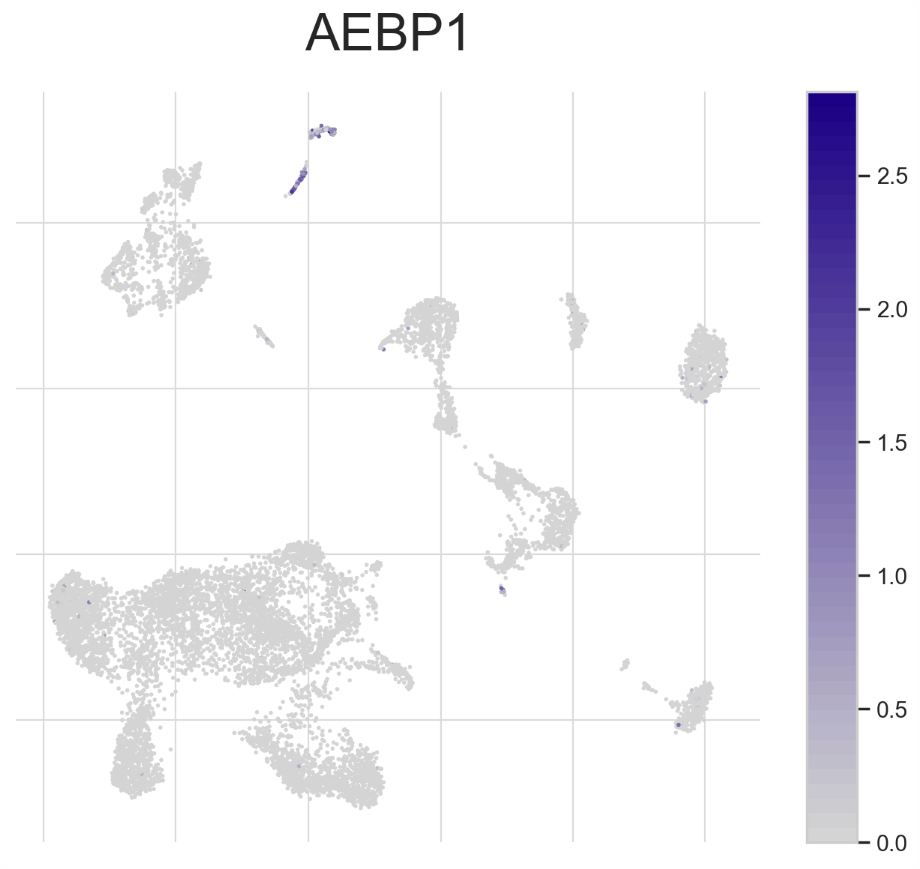

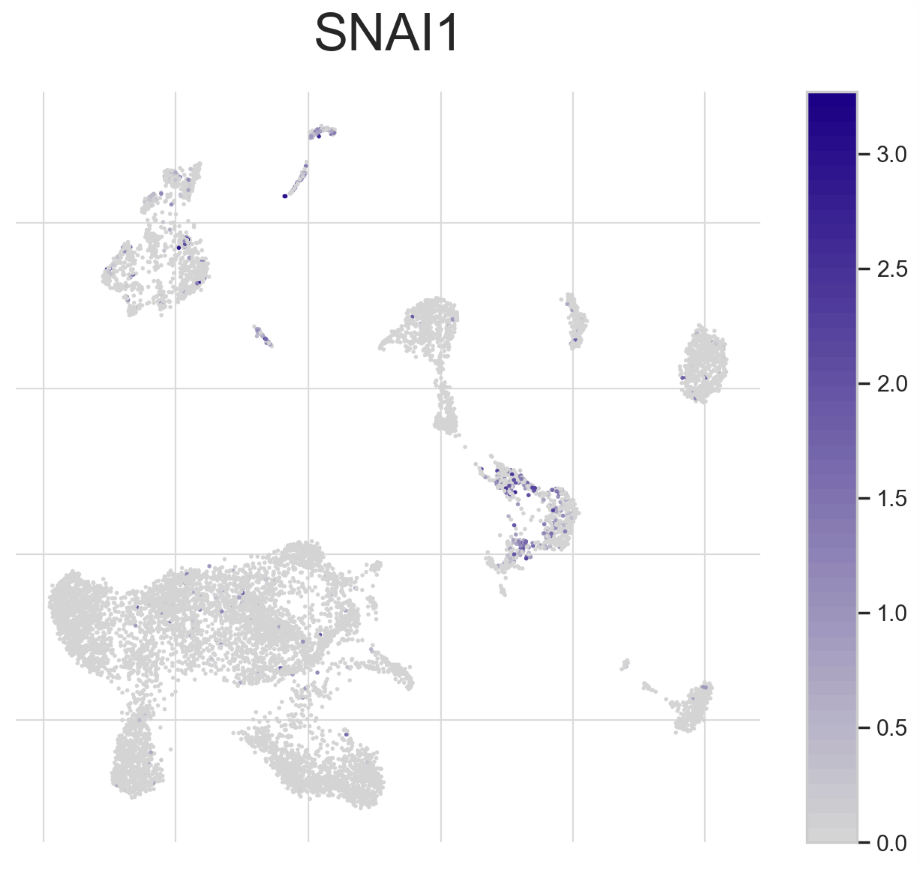

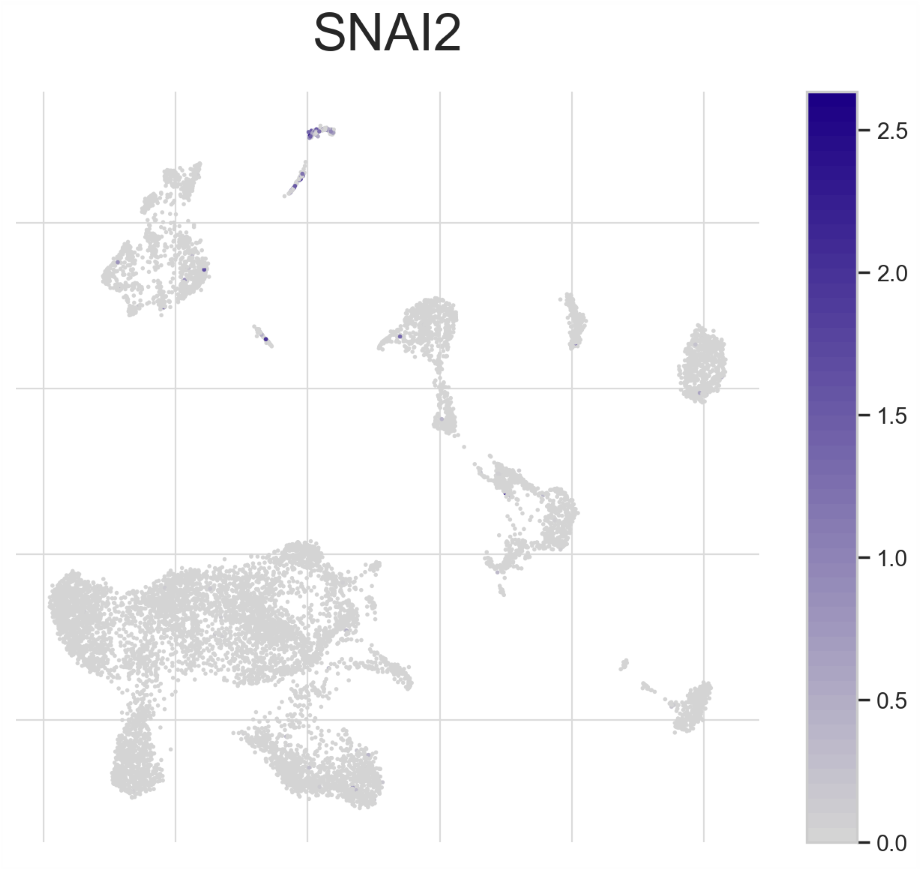

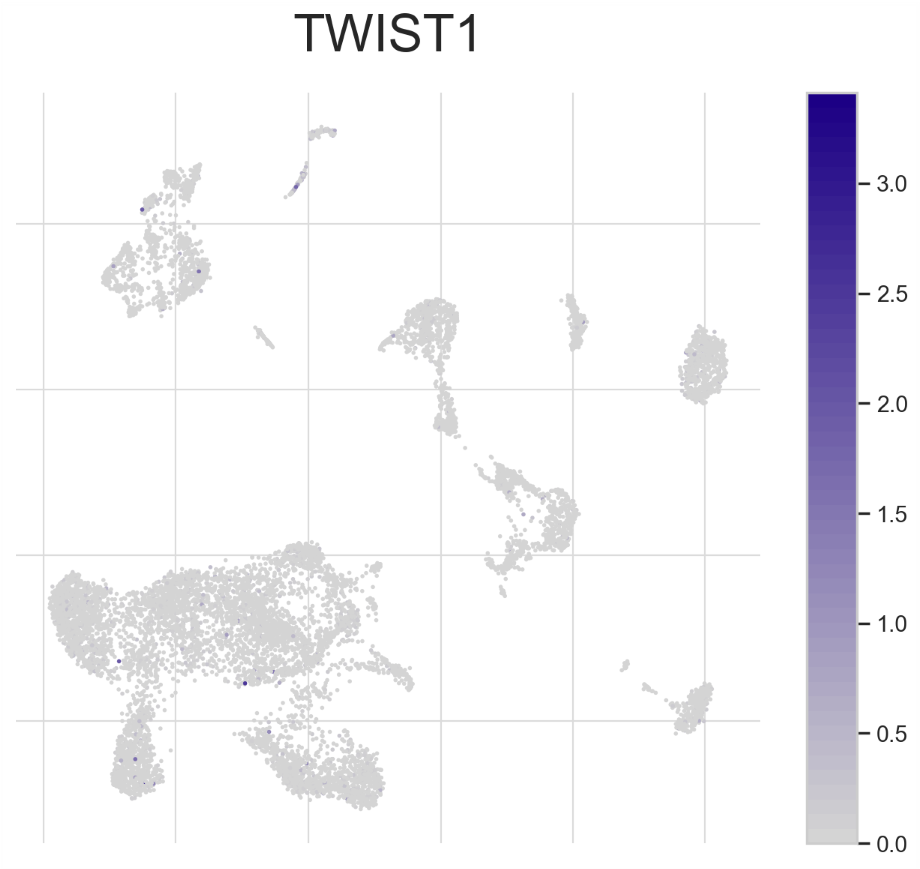

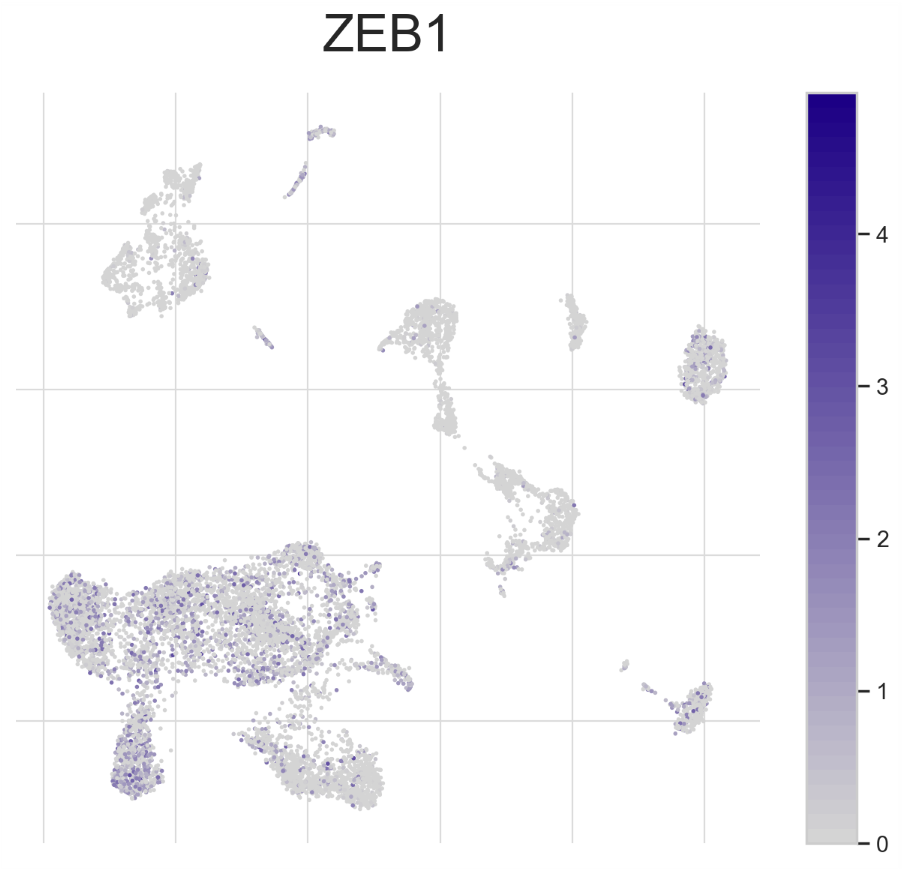

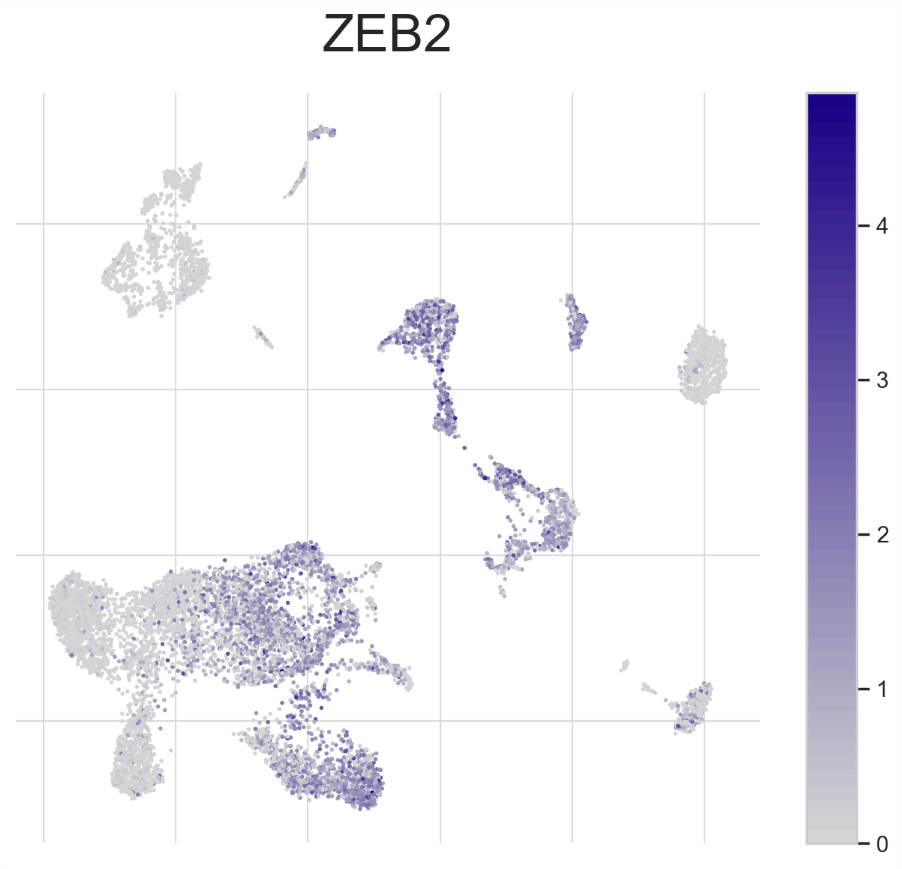


Fibroblasts

Fibroblasts

Fibroblasts

Fibroblasts

Fibroblasts

Fibroblasts

SupplementFigure2
